# Changes in the Brain Connectome Following Repetitive Transcranial Magnetic Stimulation for Stroke Rehabilitation

**DOI:** 10.7759/cureus.19105

**Published:** 2021-10-28

**Authors:** Jacky T Yeung, Isabella M Young, Stephane Doyen, Charles Teo, Michael E Sughrue

**Affiliations:** 1 Centre for Minimally Invasive Neurosurgery, Prince of Wales Private Hospital, Sydney, AUS; 2 Research, Omniscient Neurotechnology, Sydney, AUS; 3 Neurological Surgery, Prince of Wales Private Hospital, University of New South Wales, Sydney, AUS

**Keywords:** neurorehabilitation, rehabilitation, tms, connectome, stroke

## Abstract

Repetitive transcranial magnetic stimulation (rTMS) is a promising approach for post-stroke rehabilitation but there lacks a rationale strategy to plan, execute, and monitor treatment. We present a case of targeted rTMS using the Omniscient Infinitome software to devise targets for treatment in a post-stroke patient and describe the functional connectomic changes after treatment. A 19-year-old female with no medical history presented 19 months after suffering a left middle cerebral artery (MCA) superior division ischemic stroke, resulting in language impairment and diminished right upper extremity motor function. She underwent a resting-state MRI (rsMRI) with tractography and images were processed using the Omniscient Infinitome software. Analysis using the anomaly detection within the software enabled us to identify three targets for rTMS (left area 1, left area 45, and right area SFL). These areas were treated with 25 sessions of intermittent Theta Burst Stimulation (iTBS) over five days at 80% of motor threshold concomitantly with targeted physical therapy and speech therapy. At five months follow-up, her language and right upper extremity functions significantly improved. Her connectomic analysis revealed substantial neural changes, including normalization of the sensorimotor network, substantially thicker callosal fiber bundle connecting the two hemispheres, and increased cortical recruitment in her language network. We present the first description of robust connectomic alterations in a post-stroke patient following targeted rTMS treatment. Further studies on the use of rTMS with an emphasis on functional connectomics are warranted.

## Introduction

Ischemic strokes are a leading cause of disability in the world [1]. The socioeconomic impact of such a disabling disease is magnified in the younger population as these patients will carry the deficits for the rest of their lives. Intense rehabilitation can lead to some improvement in younger patients but many do not return to work due to functional limitations [2]. There is a paucity of treatment options currently for patients who have suffered ischemic strokes.

Repetitive transcranial magnetic stimulation (rTMS) has been investigated as a treatment adjunct to hasten neurorehabilition [3,4]. It is an extremely safe intervention with the most common side effects being minor dizziness, discomfort at the stimulation site, and mild headache [5]. From the robust literature derived from its use in psychiatric disorders, such as major depressive disorder, it has been demonstrated that rTMS has the potential to rewire neural networks and potentiate reorganizations of neural circuitry [6]. Recent attempts were made to utilize rTMS post-ischemic stroke to improve neurological function (extensively reviewed by Dionísio et al) [7]. The rationale for this approach is based on the concept of interhemispheric inhibition, in which the affected hemispheric experiences increased contralateral inhibition in the setting of decreased ipsilateral excitation. As such, two general options to leverage this concept are to administer rTMS (1) to the contralesional side (to decrease inhibition) or (2) to the lesional side (to increase excitability of the lesional area). A limitation to this approach is the oversimplification of the functional connectome, which has been demonstrated to contain at least 180 functional areas per hemisphere [8]. In order to rationalize the target(s) utilized in rTMS treatment for post-stroke patients, it would be prudent to be able to visualize modifiable brain areas and quantify their connectivity at a personalized level.

We report a case of rTMS used for post-stroke neurorehabilitation in a young female in conjunction with a novel, cloud-based software (Omniscient Infinitome) that provides detailed network maps of her functional connectomes. We demonstrate the utility of this software in selecting rationale rTMS targets and demonstrating changes in fiber tracts after intervention.

## Case presentation

**Figure 1:**
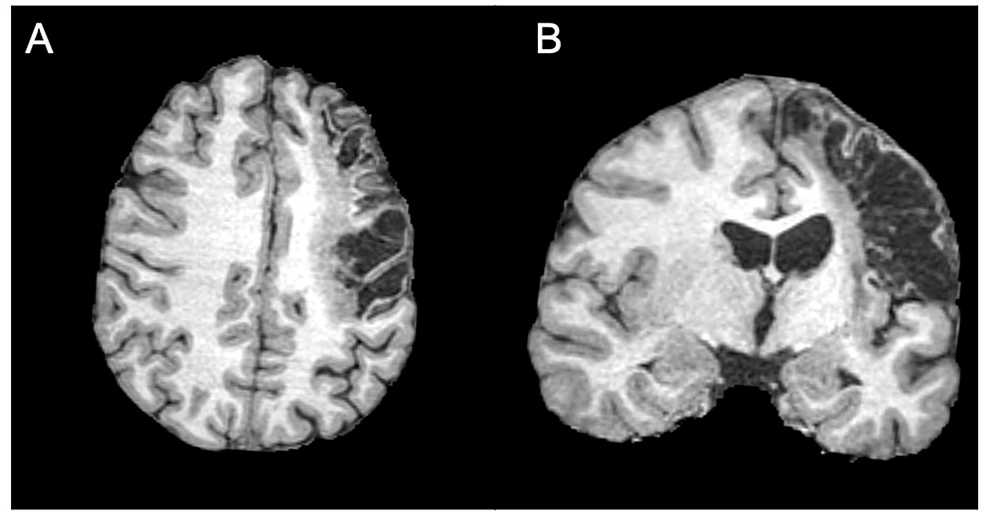
T1-weighted MRI, axial (A) and coronal (B) views, demonstrates extensive cystic encephalomalacia and gliosis in the territory of the cortical branches of the superior division of the left MCA. MCA: middle cerebral artery.

The patient was a 19-year-old right-handed female without any past medical history who suffered an ischemic stroke, resulting in extensive cystic encephalomalacia and gliosis in the territory of the cortical branches of the superior division of the left middle cerebral artery (MCA) (Figure 1). As a result, she has vast impairment of her right upper extremity, especially hand, function and her language function. She had moderate phonological text alexia, reading characterized by reduced fluency, rate, hesitations, mispronunciations and word omissions She underwent 19 months of standard rehabilitation with physical, occupational, and speech therapy after which it was felt by her and her family that her progress had plateaued. She was referred to our clinic for consideration of off-label rTMS treatment with the goal of improving her hand mobility and language functions.

**Figure 2:**
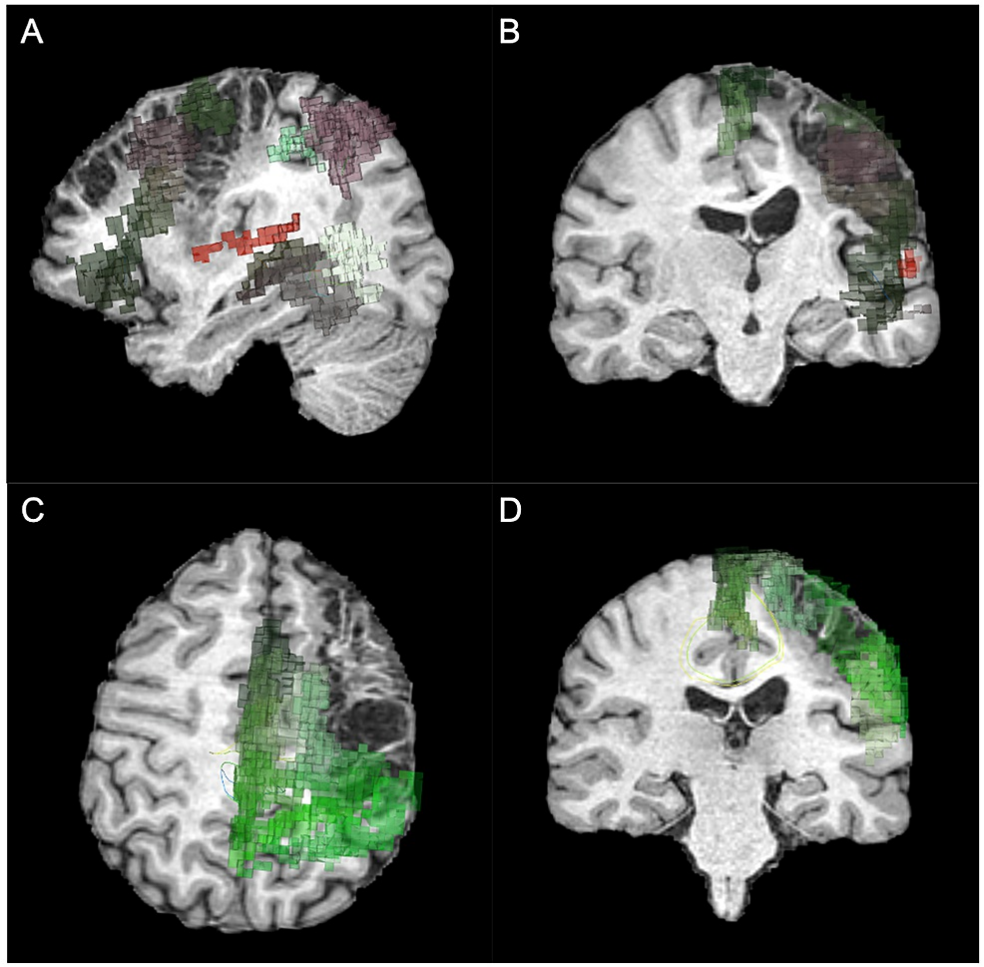
Omniscient Infinitome analysis of the stroke encompassing areas of the language network (A and B) and sensorimotor network (C and D).

**Figure 3:**
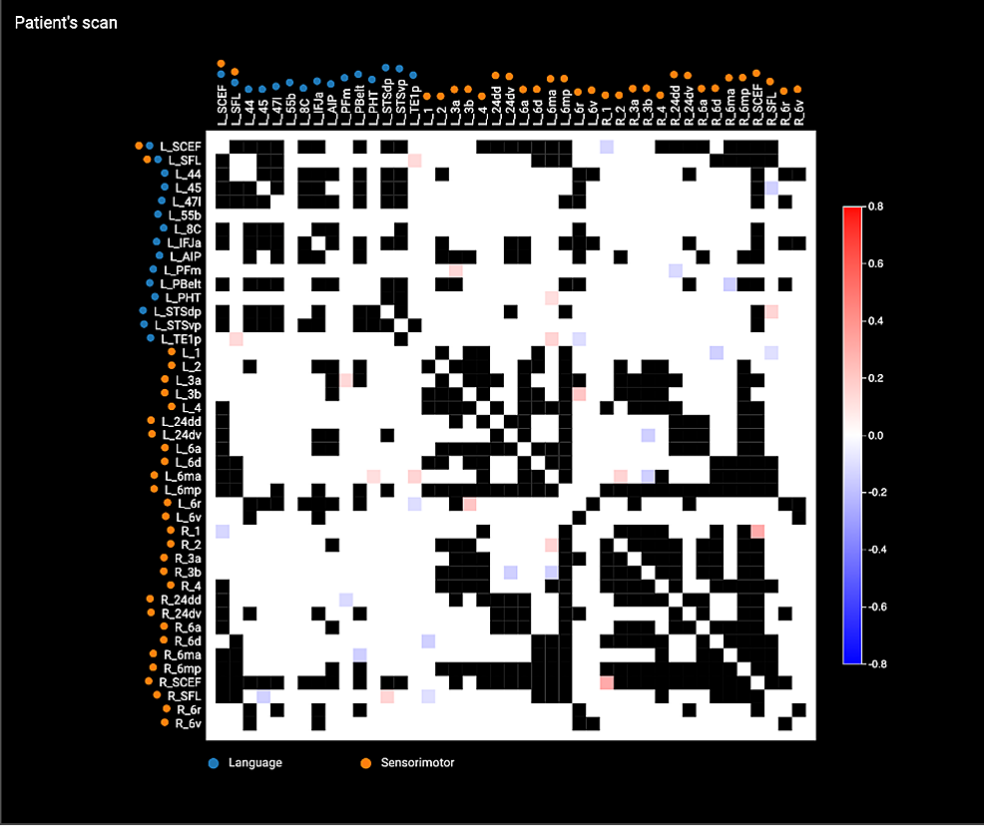
Anomaly analysis of the language and sensorimotor networks. The anomaly analysis is performed by comparing areas of the queried networks in the individual subject and their connections (number of tracts) with each other and comparing against data obtained from healthy individuals via a machine learning algorithm. The scale bar represents standard deviations with red and blue suggesting increased and decreased connectivity, respectively. Blacked-out matrices represent omitted data due to signal noise.

She underwent an MRI, consisting of 3D T1-weighted TFE, DTI and resting-state fMRI, and the DICOM images were uploaded into the Omniscient Infinitome software, which is a cloud-based program that allows DTI visualization and parcellations of various neural networks. Upon visual inspection of her neural tracts, it was clear that her stroke encompassed the frontal premotor area and the inferior frontal gyrus affecting her language network (Figure 2A and Figure 2B) and sensorimotor network (Figure 2C and Figure 2D). We utilized an anomaly detection function embedded within the software, which enabled the identification of potential areas of abnormal connectivity inferred from comparisons to normative data and registered back to the patient’s anatomy (Figure 3). We identified three targets for rTMS, including (1) left area 1 (L_1, primary motor area) to improve her hand function, (2) left area 45 (L_45), which is one of the only remaining areas of her language network that was not damaged by the stroke, and (3) right area SFL (R_SFL), which was thought to be a tract serving to support her remaining language network. These areas were treated with 25 sessions of intermittent Theta Burst Stimulation (iTBS) over five days at 80% of motor threshold [9]. The iTBS treatments were administered concomitantly with targeted physical therapy and speech therapy.

**Figure 4:**
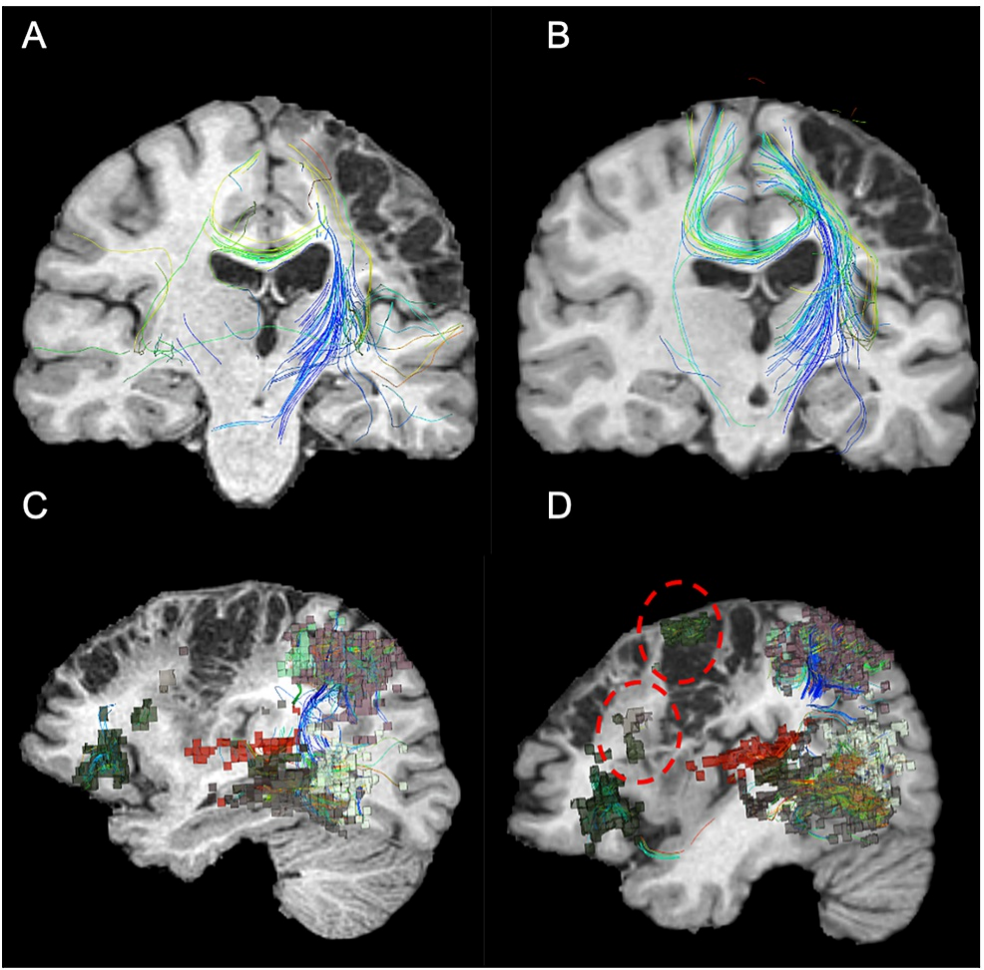
Functional connectome before and after the initial TMS treatment. There was a noticeable increase in crossing fiber bundle (A and B) involved in the left sensorimotor network before (A) and after (B) rTMS treatment. There was an increase in the frontal cortical representations (red dotted circles) detected in the language before (C) and after (D) rTMS treatment. rTMS: repetitive transcranial magnetic stimulation.

At five months follow-up, her language and right upper extremity improvements were noted by herself, her family, and the therapists. Her connectomic analysis revealed substantial neural changes, including normalization of the sensorimotor network (Figure 3B), particularly in the areas that were stimulated and her previously underactive language network now had areas of increased activity. Her left sensorimotor network that was previously supported by a handful of fibers connecting her right supplementary motor area to the left side demonstrated a substantially thicker fiber bundle connecting the two hemispheres (Figure 4A and Figure 4B). Her language network demonstrated increased cortical recruitment as visualized by the number of parcellations that were absent from the pretreatment scan (Figure 4C and Figure 4D). Her speech pathologist noted significant improvements in writing (including grammar and punctuation), improved reading (by the timing of reading the same passage before and after rTMS), fluency, speed, ability to pronounce multisyllabic words, and reduced frequency of hesitations/omissions.

**Figure 5:**
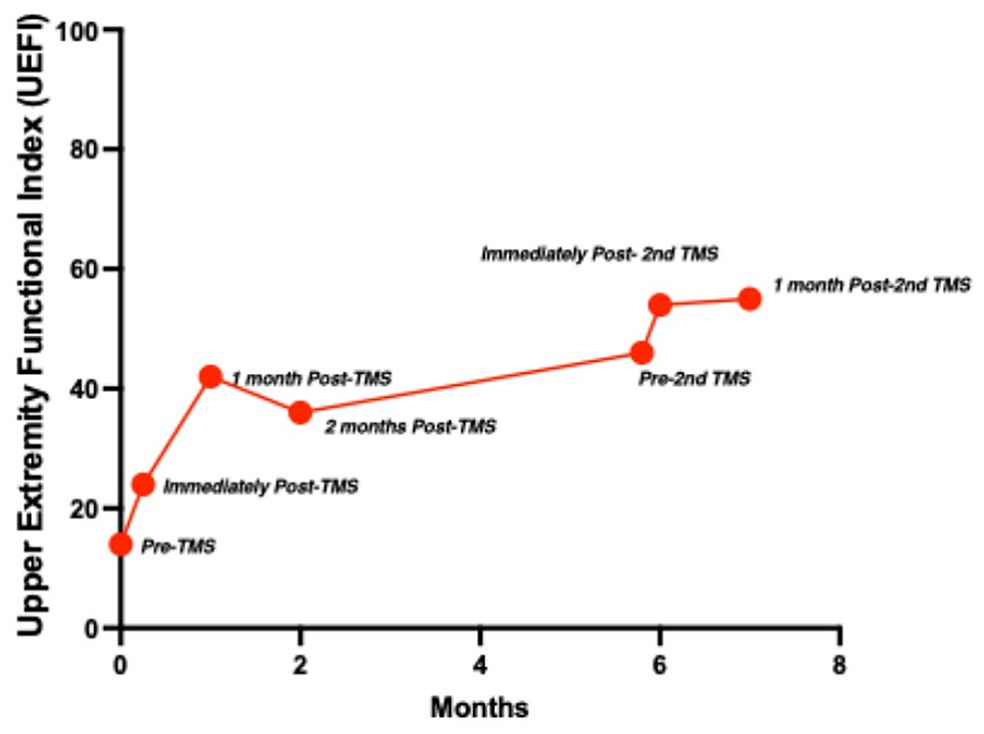
There was an overall increase in the UEFI through the course of the TMS treatments. UEFI: upper extremity functional index.

Due to the perceived improvements, the patient and her family elected to undergo another round of rTMS with L_1, R_SFL, and L_TE1p (an abnormality noted in her central executive network) chosen as targets in the same fashion using the abnormality detector with the goal to further improve her right upper extremity dexterity (functional outcome in Figure 5). 

## Discussion

Repetitive TMS has shown promise in improving functional outcomes in patients struggling to recover from ischemic strokes over conventional therapy alone [10]. In the current study, we report the use of rTMS in a post-stroke patient with the goals of improving language and her upper extremity functions after exhausting conventional rehabilitation towards the tail-end of usual post-stroke recovery [11-13]. Although previous studies have already shown that TMS can readily improve motor learning performance, cortico-excitability, and aphasia, we have demonstrated that this treatment can induce neuroplasticity and alter functional connectivity by our ability to visualize brain networks in real time [3,14-16].

Neuroplasticity associated with the recovery and associated changes in tractography have been previously demonstrated in post-stroke patients [17]. Tract integrity may even serve as a biomarker for post-stroke recovery [18]. In the current report, we not only presented the utility of tractography in monitoring recovery but we demonstrated how we leveraged our ability to visualize and quantify functional brain networks to plan, execute, and monitor rTMS treatment in this young, but functionally debilitated, patient. In our opinion, a current limitation in furthering rTMS as a treatment adjunct to post-stroke rehabilitation is due to the lack of tools to devise a rationale and scientific strategy to select appropriate brain areas for intervention. The software utilized in this case represents a usable platform for clinicians to visualize an individual patient’s personal connectome. It combines rsMRI with tractography to create a personal structural connectivity atlas, allowing us to critically evaluate aberrations in functional connectivity. We concede that target selection requires a hypothesis-driven framework - for example, improving language performance by targeting remaining, uninjured parcellations within the network (L_45) and the associated R_SFL. Although this is a single case, the post-treatment robust changes in the tracts and increased in cortical representations in the language and networks make strong suggestions that (1) neuroplasticity is present and may be inducible/accelerated with rTMS and (2) the ability to strategize individualized treatment targets may result in enhanced neurological recovery even in subacute/chronic strokes such as this case.

Outside of the confines of a single case report, we acknowledge the fact that this patient had several advantages that may have positively influenced her outcome. Aside from her young age and presumably more robust neuroplasticity, her relative independence allowed her to participate in therapy [19]. Furthermore, rTMS is useful only when there is a remaining cortex spared from ischemia that can be targeted. If the corticospinal tract or the primary motor cortex or the entire left hemisphere was destroyed in this patient, chances of recovery would conceivably be low to none. We hope that this report would encourage further studies of rTMS as a treatment adjunct for stroke rehabilitation in a systematic fashion focusing on connectomic changes.

## Conclusions

We report a case of robust connectomic alterations in a chronic post-stroke patient following rTMS treatment. It suggests that individualised iTBS treatment guided by anomaly detection algorithms could be a successful modality to improve speech and motor outcomes long-term, even in stroke patients who have seen no improvement in their condition for over a year. Further studies on the use of rTMS with an emphasis on functional connectomics are warranted.

## References

[1] (2017). Global, regional, and national burden of neurological disorders during 1990-2015: a systematic analysis for the Global Burden of Disease Study 2015. Lancet Neurol.

[2] Varona JF, Bermejo F, Guerra JM, Molina JA (2004). Long-term prognosis of ischemic stroke in young adults. Study of 272 cases. J Neurol.

[3] Wang CP, Tsai PY, Yang TF, Yang KY, Wang CC (2014). Differential effect of conditioning sequences in coupling inhibitory/facilitatory repetitive transcranial magnetic stimulation for poststroke motor recovery. CNS Neurosci Ther.

[4] Liao X, Xing G, Guo Z (2017). Repetitive transcranial magnetic stimulation as an alternative therapy for dysphagia after stroke: a systematic review and meta-analysis. Clin Rehabil.

[5] Kakuda W, Abo M, Sasanuma J (2016). Combination protocol of low-frequency rTMS and intensive occupational therapy for post-stroke upper limb hemiparesis: a 6-year experience of more than 1700 Japanese patients. Transl Stroke Res.

[6] Teneback CC, Nahas Z, Speer AM (1999). Changes in prefrontal cortex and paralimbic activity in depression following two weeks of daily left prefrontal TMS. J Neuropsychiatry Clin Neurosci.

[7] Dionísio A, Duarte IC, Patrício M, Castelo-Branco M (2018). The use of repetitive transcranial magnetic stimulation for stroke rehabilitation: a systematic review. J Stroke Cerebrovasc Dis.

[8] Glasser MF, Coalson TS, Robinson EC (2016). A multi-modal parcellation of human cerebral cortex. Nature.

[9] Talelli P, Greenwood RJ, Rothwell JC (2007). Exploring Theta Burst Stimulation as an intervention to improve motor recovery in chronic stroke. Clin Neurophysiol.

[10] Abo M, Kakuda W, Momosaki R (2014). Randomized, multicenter, comparative study of NEURO versus CIMT in poststroke patients with upper limb hemiparesis: the NEURO-VERIFY Study. Int J Stroke.

[11] Pan SL, Lien IN, Yen MF, Lee TK, Chen TH (2008). Dynamic aspect of functional recovery after stroke using a multistate model. Arch Phys Med Rehabil.

[12] Johnston M, Pollard B, Morrison V, MacWalter R (2004). Functional limitations and survival following stroke: psychological and clinical predictors of 3-year outcome. Int J Behav Med.

[13] Jørgensen HS, Reith J, Nakayama H, Kammersgaard LP, Raaschou HO, Olsen TS (1999). What determines good recovery in patients with the most severe strokes? The Copenhagen Stroke Study. Stroke.

[14] Kim YH, You SH, Ko MH (2006). Repetitive transcranial magnetic stimulation-induced corticomotor excitability and associated motor skill acquisition in chronic stroke. Stroke.

[15] Fregni F, Boggio PS, Valle AC (2006). A sham-controlled trial of a 5-day course of repetitive transcranial magnetic stimulation of the unaffected hemisphere in stroke patients. Stroke.

[16] Dionísio A, Duarte IC, Patrício M, Castelo-Branco M (2018). Transcranial magnetic stimulation as an intervention tool to recover from language, swallowing and attentional deficits after stroke: a systematic review. Cerebrovasc Dis.

[17] Kierońska S, Świtońska M, Meder G, Piotrowska M, Sokal P (2021). Tractography alterations in the arcuate and uncinate fasciculi in post-stroke aphasia. Brain Sci.

[18] Lindenberg R, Zhu LL, Rüber T, Schlaug G (2012). Predicting functional motor potential in chronic stroke patients using diffusion tensor imaging. Hum Brain Mapp.

[19] Burke SN, Barnes CA (2006). Neural plasticity in the ageing brain. Nat Rev Neurosci.

